# Balsam of Peru in the Treatment of Scabies

**Published:** 1907-03-16

**Authors:** 


### Points in Treatment.

### BALSAM OF PERU IN THE TREATMENT OF SCABIES.

The treatment of scabies, or "itch," is not as a rule very difficult when the patient is intelligent; one who is ordinarily clean, but who has accidentally become infected with the *sarcoptes hominis* may be cured with comparative ease. It is amongst the uncleanly, and therefore, for the most part, amongst the lower classes, that treatment is liable to be unsuccessful.

The treatment in all cases must be directed to ridding the patient of the parasite, and to this end both the person and the clothes require attention. In a cleanly patient who has facilities for taking baths repeatedly, the usual course is as follows: A very hot bath is prepared, the patient soaks in it for half an hour or more, all clothes that have been worn during the infection are taken away, and, in the case of a rich patient, destroyed; in cases where this cannot be afforded the clothes may be disinfected in a Washington-Lyon apparatus if it is available, or else by boiling in the case of under-clothing, and baking in the case of such articles as would be damaged by boiling. After half-an-hour's soaking in the very hot bath, the patient washes himself all over with plenty of soft soap and scrubs himself, especially over the affected parts, with an old scrubbing-brush, the bristles of which have become softened from frequent use. In this way the epithelium over many of the burrows of the female parasites becomes thinned sufficiently for the remedies applied subsequently to reach the parasites themselves and their ova. At the same time it is unwise to use too hard a scrubbing-brush, else the skin may become actually excoriated and so sensitive that acute dermatitis may follow. After soaping and scrubbing, and removing the soap by a second immersion in the bath, the patient is thoroughly dried with a clean towel and one or other of the parasiticidal ointments mentioned below is applied all over the affected parts and over a considerable area around them. It is well not only to smear the ointment directly on to the skin, but also to spread some more of it on lint and to apply the ointment side of the lint to the affected parts, keeping the lint on with bandages. A clean night-shirt is then put on, and the patient goes to bed. Meanwhile the scrubbing-brush that was used is disinfected by boiling, the remains of the cake of soap is thrown away; next day the nightshirt that has been worn and the bed-linen should be boiled. The patient may get up next day, but anything that he wears must be such as can be either disinfected or destroyed afterwards, and it must not have been worn, without disinfection, since the infection with scabies began. Diet is, of course, as usual. It is best for the ointment, lint, and bandage to be kept on all that day until the evening. Then another hot bath is given, the soaping, scrubbing, drying, and renewal of the ointment being repeated as before. The soap should be a fresh cake; the scrubbing-brush and towel are either fresh ones, or the former ones disinfected; fresh lint and fresh bandage, a fresh nightgown, and fresh sheets are used; and so on till the third night, when the bath, soaping, scrubbing, and ointment are repeated a third time. It is probable that every "run" will have been completely exposed to the ointment by this time, so that on the fourth day the ointment may be omitted, the patient dressing in the usual way, and, if the treatment has been thorough in all its minutiæ, he may return to daily work quite cured.

In some cases there is a great deal of pus-inoculation of the skin in addition to the scabies proper. It will then be necessary to treat the impetigo first, either by hot boric acid fomentations or by unguentum hydrargyri oxidi flavi (B.P.); dealing with the scabies subsequently by the method detailed above.

For the scabies itself, the parasiticidal ointment usually employed is one or other of the following three:—

1. Unguentum staphisagriæ (B.P.), which is made from stavesacre seeds, 4 parts; yellow beeswax, 2 parts; benzoated lard, 17 parts.
2. Unguentum styracis, prepared as follows: take of prepared storax, 145 grains; of methylated spirit, 3 fluid drachms; and of lard, 1 ounce; dissolve the storax in the spirit and mix with the lard.
3. Unguentum sulphuris (B.P.), which is made from sublimed sulphur, 1 part; benzoated lard, 9 parts.

Perhaps the unguentum styracis is the best of the three.

It will be seen that great care is needed, also a great deal of trouble, and at least three good hot baths on successive nights to ensure a cure. With poorer people it is next to impossible to carry out all the details of this treatment thoroughly, and it would be a great thing if we knew of a treatment which did not necessitate so many baths. Lieut.-Colonel S. C. B. Robinson, of the Royal Army Medical Corps, describes such a method, in which not merely is it only necessary to give one bath, but further than this it appears to be absolutely essential that no further bath should be given for at least four weeks. He has found it most efficacious amongst soldiers. The patient is given a hot bath, followed by soaping and scrubbing, as described above. He is then dried quickly and very thoroughly: the affected parts and a surrounding area of healthy skin are then rubbed over with the following preparation: balsam of peru, 3 parts; glycerine, 1 part.

The balsam must be well rubbed into flexures and crevices; and allowed to dry in as far as it will. There is no absolute necessity to dress the parts with lint and bandage; the patient may dress himself and go about his work, provided always that he be not allowed to wear any clothes he has worn since he became infected, until these have been thoroughly disinfected. A striking result of the treatment is *the immediate cessation of the itching* after the first application; the itching has sometimes been so extreme that sleep was impossible; ability to sleep immediately follows the treatment. Next day, some more of the balsam of peru and glycerine may be applied, and similarly on the third day; but it is stated that one application is usually enough by itself. The cases which fail are those in which repeated baths are taken; there is little doubt, therefore, that the treatment with unguentum styracis will always be preferable amongst the better classes; but amongst the poor of cities it is a boon to have some method of treatment in which a single bath will suffice; this is the main advantage of the balsam of peru treatment, the essential points to ensure success with which are:—

1. That the patient must be thoroughly washed and scrubbed at the commencement.

2. That the preparation must be well and conscientiously rubbed into the skin all over immediately after the patient has been dried.

3. That no bath must on any account be taken till four weeks after the first application.

